# Insights into the binding mode of sulphamates and sulphamides to hCA II: crystallographic studies and binding free energy calculations

**DOI:** 10.1080/14756366.2017.1349764

**Published:** 2017-07-24

**Authors:** Giuseppina De Simone, Emma Langella, Davide Esposito, Claudiu T. Supuran, Simona Maria Monti, Jean-Yves Winum, Vincenzo Alterio

**Affiliations:** a Istituto di Biostrutture e Bioimagini, Consiglio Nazionale delle Ricerche, Naples, Italy;; b Neurofarba Department, Section of Pharmaceutical and Nutriceutical Sciences, Università degli Studi di Firenze, Sesto Fiorentino, Florence, Italy;; c Institut des Biomolécules Max Mousseron, UMR 5247 CNRS, ENSCM, Université de Montpellier, Montpellier, France

**Keywords:** Carbonic anhydrase, crystal structure, binding free energy calculations

## Abstract

Sulphamate and sulphamide derivatives have been largely investigated as carbonic anhydrase inhibitors (CAIs) by means of different experimental techniques. However, the structural determinants responsible for their different binding mode to the enzyme active site were not clearly defined so far. In this paper, we report the X-ray crystal structure of hCA II in complex with a sulphamate inhibitor incorporating a nitroimidazole moiety. The comparison with the structure of hCA II in complex with its sulphamide analogue revealed that the two inhibitors adopt a completely different binding mode within the hCA II active site. Starting from these results, we performed a theoretical study on sulphamate and sulphamide derivatives, demonstrating that electrostatic interactions with residues within the enzyme active site play a key role in determining their binding conformation. These findings open new perspectives in the design of effective CAIs using the sulphamate and sulphamide zinc binding groups as lead compounds.

## Graphical Abstract

**Figure:**
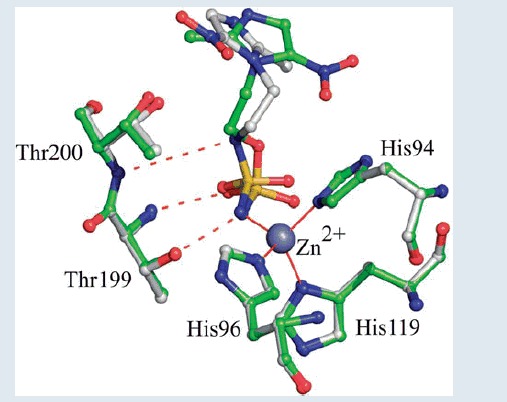

## Introduction

Carbonic anhydrases (CAs; EC: 4.2.1.1) are a family of metalloenzymes present in all kingdoms of life that catalyse the interconversion of carbon dioxide and bicarbonate^1^. Based on their structural features, they are grouped into seven different classes, namely α-, β-, γ-, δ-, ζ-, η- and θ-CAs. α-CAs are predominantly expressed in vertebrates, bacteria, algae and cytoplasm of green plants, β-CAs in bacteria, algae and chloroplasts, γ-CAs in archaea and some bacteria, δ- and ζ-CAs in some marine diatoms, η-CAs only in the protozoan parasite *Plasmodium* spp., whereas the recently discovered θ-class has been so far found only into the marine diatom *Phaeodactylum tricornutum*^1–8^. Humans encode 12 catalytically active α-CA isozymes, which differ in molecular features, oligomeric arrangement, kinetic properties and cellular localisation, with isoforms I, II, III, VII and XIII localised in the cytosol, CA IV, IX, XII and XIV associated with the cell membrane, CA VA and VB confined in mitochondria, and CA VI secreted in saliva and milk^1^. All catalytically active human (h) CAs contain in the active site a Zn^2+^ ion essential for catalysis; this ion is coordinated by three conserved histidine residues (His94, His96 and His119) and a water molecule/hydroxide ion^1^. hCAs participate in several physiological processes, among which pH homeostasis, CO_2_ and HCO_3_^−^ transport, cell differentiation and proliferation, respiration, bone resorption, neurotransmission, ureagenesis, gluconeogenesis, lipogenesis, and fertilisation^9^^,^^10^. Abnormal levels and/or activities of these enzymes have been often associated with different human diseases, such as glaucoma, epilepsy, high-altitude sickness, as well as cancer^11^. For these reasons, hCAs represent an important target for the design of inhibitors or activators with biomedical applications^11^^,^^12^.

**Figure 1.**
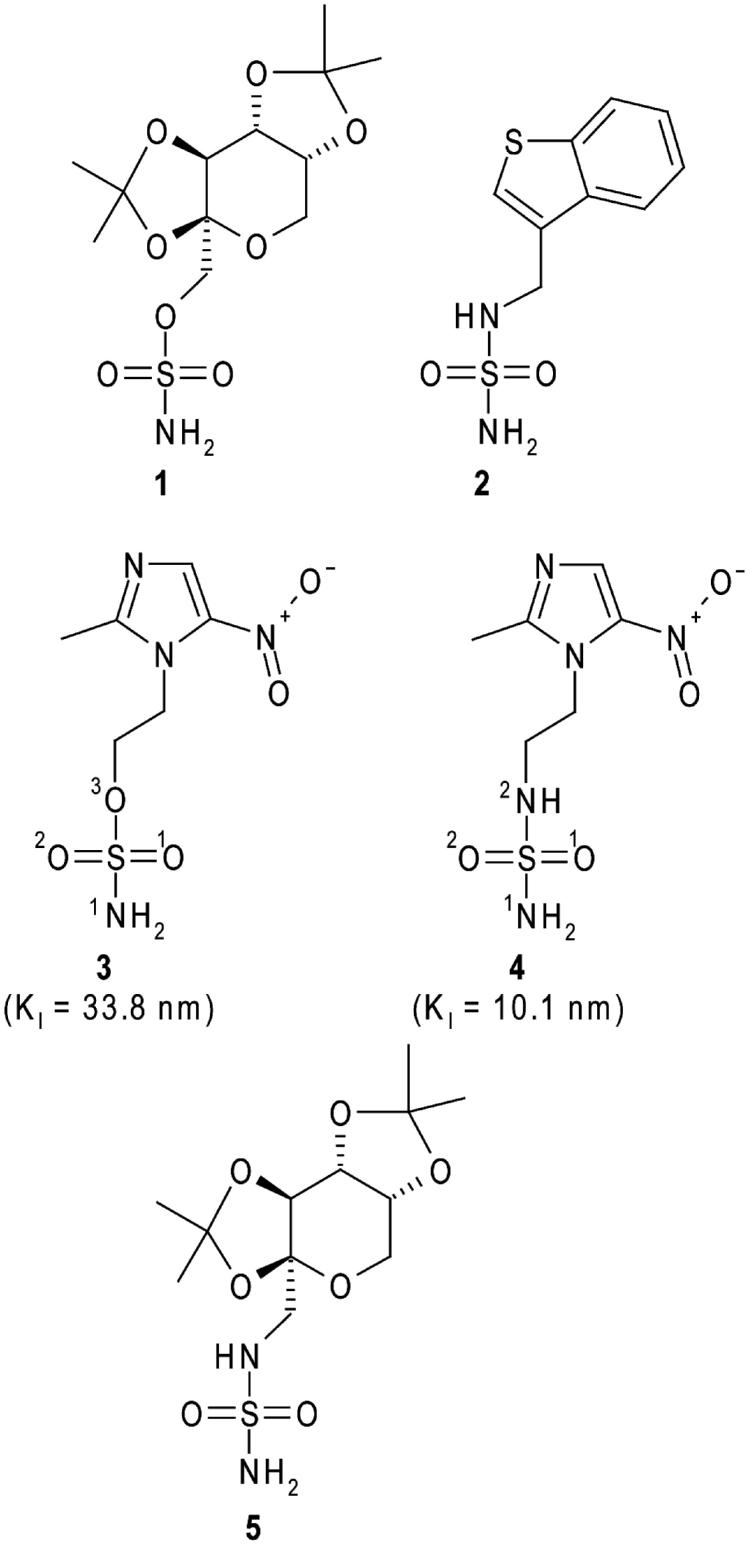
Structural formulas of topiramate **(1)**, JNJ-26990990 **(2)**, 2-methyl-5-nitro-imidazole-sulphamate **(3)**, 2-methyl-5-nitro-imidazole-sulphamide **(4)** and the topiramate sulphamide analogue **(5)**. hCA II inhibition constants for compounds **3** and **4** are also reported^26^.

The most studied carbonic anhydrase inhibitors (CAIs) are sulphonamide derivatives (R-SO_2_NH_2_), which are able to bind in a tetrahedral geometry the active site zinc ion, substituting the water molecule/hydroxide ion present in the native enzyme^1^. These molecules have been largely investigated, due to their capability to strongly bind to the hCA active site, with many such agents in clinical use^11^^,^^13^; however, the occurrence of various undesired side effects due to the lack of selectivity against the different CA isoforms strongly limits their use as drugs^1^^,^^11^. Therefore, other CAI classes with different zinc-binding groups (ZBGs) have been developed over the years, with sulphamates (R-O-SO_2_NH_2_) and sulphamides (R-NH-SO_2_NH_2_) among the most important ones. These compounds differ from sulphonamides for the additional presence of an electron withdrawing group, an oxygen atom in the case of sulphamates^14^ and an NH group in the case of sulphamides^15^. As observed for sulphonamides, also sulphamates and sulphamides exert their inhibitory action through coordination to zinc ion and consequent substitution of the water molecule/hydroxide ion^1^. Plenty of studies has been reported showing that many sulphamates possess effective inhibitory properties against all known human isoforms^1^^,^^11^^,^^16–19^, with some derivatives, such as the sugar sulphamate topiramate (compound **1** in Figure 1), successfully used for the treatment of a variety of diseases such as epilepsy, migraine, and obesity^20^^,^^21^. Although the sulphamide group was initially considered not particularly suitable for obtaining potent CAIs^22^, several compounds containing a primary sulphamide moiety have also been proved to possess a high CA inhibition activity^1^^,^^11^^,^^19^^,^^23^. As an example, compound JNJ-26990990 (**2**) (see Figure 1), which presents excellent anticonvulsant activity and can be potentially used in the treatment of multiple forms of epilepsy, is also a nanomolar inhibitor of several CA isoforms^24^^,^^25^.

We recently reported the synthesis of a series of sulphonamide/sulphamide/sulphamate derivatives incorporating nitroimidazole moieties^26^. Inhibition studies against isoforms I, II, IX, and XII showed that these compounds, in particular, the sulphamate/sulphamide derivatives **3** and **4** (Figure 1), are good CAIs, with *K_I_* values in the nanomolar range. Moreover, compound **4** was demonstrated to inhibit *in vitro* the hypoxia-induced extracellular acidosis in two cell lines overexpressing CA IX and to enhance *in vivo,* in co-treatment with doxorubicin, sensitisation towards radiotherapy and chemotherapy of CA IX containing tumours^26^. The X-ray crystal structure of the hCA II/**4** adduct was also reported, highlighting the principal interactions responsible for the binding of the inhibitor to the enzyme active site^26^.

Within a research project aimed at understanding at the atomic level, the inhibition properties of sulphamate/sulphamide CAIs, here we report the X-ray crystal structure of the hCA II/**3** adduct and compare it with the previously obtained hCA II/**4** structure. Surprisingly, even if the two inhibitors differ for only one atom (see Figure 1), they adopt a completely different binding mode within the CA II active site. Binding free energy calculations have been used to rationalise this result.

## Materials and methods

### Crystallisation, X-ray data collection, and refinement

Crystals of the hCA II/**3** complex were prepared by soaking hCA II 100K crystals (obtained using the hanging drop vapour diffusion technique) for 1 h in the crystallisation solution (1.3 M sodium citrate, 100 mM Tris-HCl, pH 8.5) saturated with the inhibitor. Prior to X-ray data collection, crystals of the complex were transferred from the drops to a cryoprotectant solution prepared by the addition of 20% glycerol to the precipitant solution and then flash-cooled to 100K in a nitrogen stream. A complete dataset was collected at 1.80 Å resolution from a single crystal, at 100 K, with a copper rotating anode generator developed by Rigaku and equipped with Rigaku Saturn CCD detector.

Diffraction data were indexed, integrated and scaled using the HKL2000 software package^27^. A total of 107,169 reflections were measured and reduced to 22,183 unique reflections. Crystal parameters and relevant X-ray data collection statistics can be found in Table 1. Initial phases were calculated using hCA II crystallised in the P2_1_ space group (PDB code 1CA2)^28^ as starting model after deletion of non-protein atoms. An initial round of rigid body refinement followed by simulated annealing and individual B-factor refinement was performed using the programme Crystallography and NMR system (CNS)^29^^,^^30^. Model visualisation and rebuilding were performed using the graphics programme O^31^. After an initial refinement, limited to the enzyme structure, a model for the inhibitor was easily built and introduced into the atomic coordinates set for further refinement. Crystallographic refinement was carried out against 95% of the measured data. The remaining 5% of the observed data, which was randomly selected, was used for R_free_ calculations to monitor the progress of refinement. Restraints on inhibitor bond angles and distances were taken from the Cambridge Structural Database^32^, whereas standard restraints were used on protein bond angles and distances throughout refinement. Water molecules were built into peaks >3σ in |Fo| − |Fc| maps that demonstrated appropriate hydrogen-bonding geometry. Several alternate cycles of refinement and manual model building were performed to reduce the R_work_ and R_free_ to the final values of 0.157 and 0.195, respectively. Relevant refinement statistics can be found in Table 1. The refined model contained 2055 protein atoms, 237 waters, and one inhibitor molecule. Coordinates and structure factors have been deposited with the Protein Data Bank (accession code 5O07).

**Table 1.** Data collection and refinement statistics. Values in parentheses refer to the highest resolution shell (1.86–1.80 Å).

Crystal parameters	
Space group	P2_1_
a (Å)	42.2
b (Å)	41.3
c (Å)	71.7
γ (°)	104.3
Number of independent molecules	1
Data collection statistics	
Resolution (Å)	25.3–1.80
Wavelength (Å)	1.54178
Temperature (K)	100
*R*_merge_ (%)^a^	3.5 (9.1)
<I>/<σ(I)>	35.8 (10.6)
Total reflections	107,169
Unique reflections	22,183
Redundancy (%)	4.8 (2.7)
Completeness (%)	98.8 (92.9)
Refinement	
Resolution (Å)	25.3–1.80
*R*_work_ (%)^b^	15.7
*R*_free_ (%)^b^	19.5
RMSD from ideal geometry	
Bond lengths (Å)	0.012
Bond angles (°)	1.7
Number of protein atoms	2055
Number of water molecules	237
Number of inhibitor atoms	16
Average B factor (Å^2^)	
All atoms	13.3
Protein atoms	12.1
Inhibitor atoms	16.0
Water molecules	23.2

a R_merge_ = Σ_hkl_Σ_i_|I_i_(hkl) − <I(hkl)>|/ Σ_hkl_Σ_i_I_i_(hkl), where I_i_(hkl) is the intensity of an observation and < I(hkl)> is the mean value for its unique reflection; summations are over all reflections.

b R_work_ = Σ_hkl_ǁFo(hkl)| − |Fc(hkl)ǁ/Σ_hkl_|Fo(hkl)| calculated for the working set of reflections. R_free_ is calculated as for R_work_, but from 5% of the data that was not used for refinement.

### Computational study

#### Systems preparation

**Complex_O** and **complex_N** models were built from the hCA II/**3** and hCA II/**4**^26^ crystallographic structures, by replacing the 2-methyl-5-nitro-imidazole moiety of the two inhibitors with a methyl group. The third model, namely **complex_NO**, was obtained by substituting the N2 atom of **complex_N** with an oxygen atom. Hydrogen atoms were added to all the models and their positions were energy minimised by 500 steps of Conjugate Gradient using the Discover module of InsightII package (Insight2000, Accelrys, San Diego, CA).

The partial atomic charges for ligands and zinc ion were obtained by quantum mechanical (QM) calculations (B3LYP/6–31 G*) using the Gaussian09 software^33^ via the Restrained ElectroStatic Potential (RESP) fitting procedure as implemented in the PyRED server^34^^,^^35^. The charges calculations were performed on model systems including the ligand, the zinc ion and the side chains of the three coordinating histidine residues. Since literature data suggest that the sulphamate and sulphamide groups, similarly to sulphonamides^36^^,^^37^, bind the zinc ion in a deprotonated form, the total charge for ligands was set at −1 e. A charge of 1.5 e was obtained for the zinc ion, whereas a high negative charge was derived for the deprotonated nitrogen atom N1 (∼ −1.7 e) in all the three ligands. A complete list of the partial charges computed for the ligands atoms is reported in Table 2. The General AMBER force field^38^, and the AMBERff14SB force field^39^ were used for the ligands and proteins, respectively. Van der Waals parameters for the Zn^2+^ ion were adopted from the work of Li et al.^40^ (σ = 1.271; ɛ (kcal/mol) = 0.00330286).

**Table 2.** Partial atomic charges (e) computed for the three ligands in **complex_O**, **complex_N** and **complex_NO**, respectively. Charges were calculated via the RESP fitting procedure as implemented in the PyRED server using Gaussian09 software.

Complex_O		Complex_N		Complex_NO	
Ligand atom	Charge	Ligand atom	Charge	Ligand atom	Charge
N1	−1.7264	N1	−1.7369	N1	−1.6903
H1	0.6300	H1	0.5896	H1	0.5976
S1	1.2394	S1	1.4216	S1	1.3758
O1	−0.4723	O1	−0.5319	O1	−0.4851
O2	−0.5586	O2	−0.5903	O2	−0.6095
O3	−0.3736	N2	−0.7886	O3	−0.4657
C1	0.0398	H2	0.4052	C1	0.3176
H11	0.1055	C1	0.2330	H11	0.0483
H12	0.0678	H11	0.0232	H12	−0.0588
H13	0.0484	H12	−0.0722	H13	−0.0300
		H13	0.0473		

#### Binding free energy calculations

The binding free energies (Δ*G*_bind_ in kcal/mol) were calculated using the Molecular Mechanics/Generalised Born Surface Area (MM/GBSA) method^41^^,^^42^ implemented in AmberTools14^43^. Moreover, to identify the key protein residues responsible for the ligands binding process, the binding free energy was decomposed on a per-residue basis.

For each complex, the binding free energy of MM/GBSA was estimated as follows:

$$\Delta G_{\text{bind}}\;=\;G_{\text{complex}}-G_{\text{protein}}-G_{\text{ligand}}$$

where ΔG_bind_ is the binding free energy and *G*_complex_, *G*_protein_ and *G*_ligand_ are the free energies of complex, protein, and ligand, respectively. The energies were estimated as shown below:

$$\Delta G_{\text{bind}}=\Delta E_{\text{gas}}+\Delta G_{\text{sol}}-\text{T}\Delta S$$

If ligands have similar structures and binding modes, it is acceptable to exclude the entropy contribution (–TΔ*S*) in practise^42^^,^^44^^,^^45^. Then the binding free energy is evaluated by^46^:

$$\begin{array}{l} \Delta G_{\text{bind}}=\Delta E_{\text{gas}}+\Delta G_{\text{sol}}\\ \Delta E_{\text{gas}}=\Delta E_{\text{MM}}=\Delta E_{\text{elec}}+\Delta E_{\text{vdW}}\\ \Delta G_{\text{sol}}=\Delta G_{\text{GB}}+\Delta G_{\text{SA}} \end{array}$$

where Δ*E*_gas_, the complete gas phase force field energy, is the molecular mechanics (MM) part Δ*E*_MM_, including van der Waals (Δ*E*_vdW_) and electrostatic (Δ*E*_elec_) contributions; Δ*G*_sol_ is the solvation free energy, and is the sum of electrostatic (Δ*G*_GB_) and non-polar (Δ*G*_SA_) interactions. The electrostatic solvation free energy (Δ*G*_GB_) is evaluated via Generalised Born implicit solvation model^47^, and the non-polar solvation free energy (Δ*G*_SA_) is estimated by the Linear Combination of Pairwise Overlaps (LCPO) method^48^.

## Results and discussion

**Figure 2.**
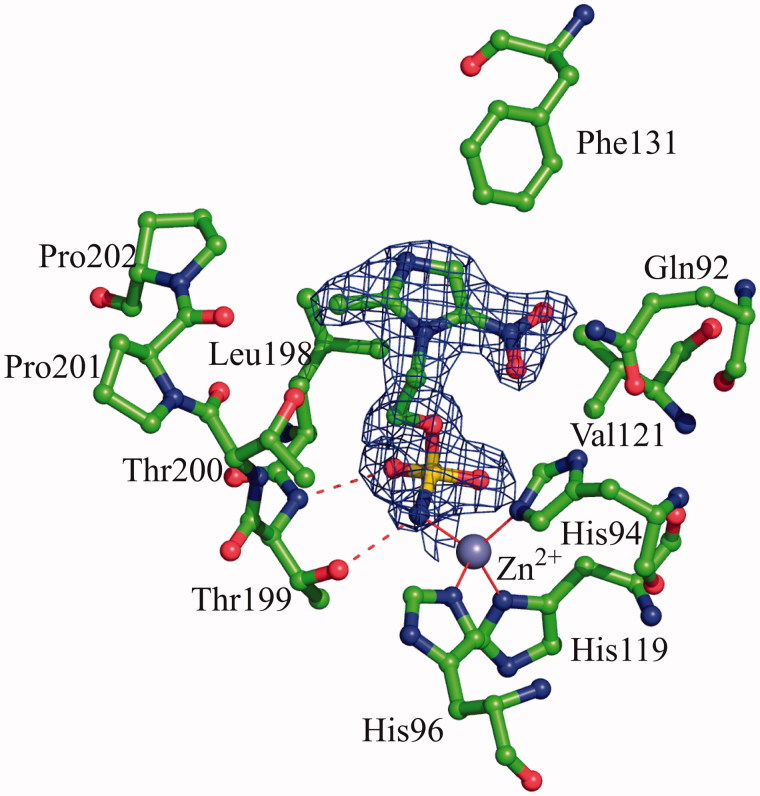
Active site region in the hCAII/**3** complex. Hydrogen bonds, active site Zn^2+^ coordination and residues establishing van der Waals interactions (distance <4.0 Å) with the inhibitor are reported. Sigma-A weighted |2Fo-Fc| simulated annealing omit map (at 1.0 sigma) relative to the inhibitor molecule is also shown.

Crystal structure of hCA II in complex with compound **3** was determined at 1.80 Å resolution, revealing a clear electron density for the inhibitor molecule in the enzyme active site (Figure 2). The model was refined with CNS^29^^,^^30^, giving final R_work_ and R_free_ values of 15.7% and 19.5%, respectively. The average B factors were 12.1 Å^2^ for the protein, 23.2 Å^2^ for the solvent and 16.0 Å^2^ for the inhibitor molecule. Data collection and refinement statistics are shown in Table 1.

The binding of the inhibitor to hCA II did not generate major changes in the protein structure as proved by the low value of the r.m.s.d. calculated by superimposing the Cα atoms in the adduct and the non-inhibited enzyme (0.3 Å). Similarly to what previously observed for other hCA II/sulphamate complexes solved so far^49–65^, compound **1** interacts directly with the zinc ion of the active site, with its sulphamate nitrogen atom N1 (for atom numbering see Figure 1) displacing the water molecule/hydroxide ion, which in the not-inhibited enzyme occupies the fourth coordination position. Additional hydrogen bonds between the sulphamate moiety and residues within the enzyme active site contribute to stabilise the binding. In detail, the sulphamate nitrogen atom N1 donates a hydrogen bond to the Thr199OG1 atom, whereas one of the two sulphamate sp^2^ oxygens accepts another hydrogen bond from the main chain nitrogen of the same residue (Figure 2). No other polar interactions were observed between the inhibitor and enzyme residues, but a large number of van der Waals contacts were present, with the imidazole ring being located in the middle of the active site cavity and the nitro group being oriented towards the hydrophilic region of it (Figure 2)^66^.

**Figure 3.**
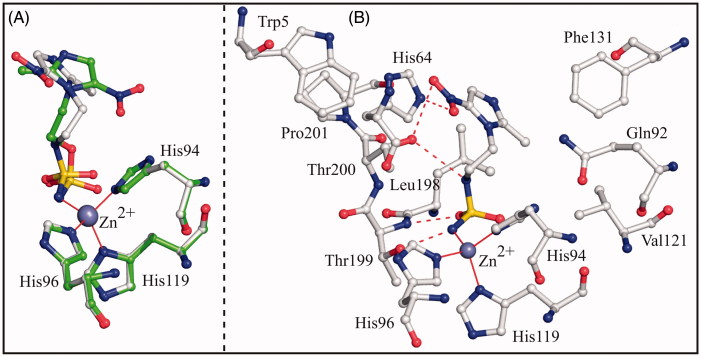
(A) Structural superposition between hCA II/**3** (green) and hCA II/**4** (white, PDB code 4MO8)^26^. (B) Active site region in the hCAII/**4** complex. Hydrogen bonds, active site Zn^2+^ coordination and residues establishing van der Waals interactions (distance <4.0 Å) with the inhibitor are reported.

To compare the binding mode of compounds **3** and **4** to the hCA II active site, the crystallographic structures of the hCA II/**3** and hCA II/**4** adducts were superimposed showing that the two inhibitors adopt a completely different binding mode to the enzyme (Figure 3(A)). Main differences were observed in the orientation of the imidazole rings, which were rotated of about 140° in the two complexes (Figure 3(A)). Because of the different orientation, inhibitor **4** established a higher number of favourable interactions with active site residues (Figure 3(B)), thus explaining its higher affinity for the enzyme (see *K_I_* values in Figure 1). Since compounds **3** and **4** differ only for one atom (O3 instead of N2) in their ZBG (see Figure 1), the structural basis of the different orientation of the imidazole rings in the active site cavity should be searched in the interactions that this atom can establish with neighbouring residues within the active site cavity. In the hCA II/**4** complex, the nitrogen atom N2 is at 3.2 Å from the Thr200OG1 atom; this distance being compatible with the formation of a weak hydrogen bond interaction. On the contrary, in the hCA II/**3** complex, the distance between the sulphamate oxygen O3 and the Thr200OG1 atom becomes of 4.7 Å. This slide away causes the rearrangement of the imidazole ring within the active site and the loss of the hydrogen bond interactions between the nitroimidazole moiety and residues His64 and Thr200.

To understand if the different position assumed by N2 and O3 atoms in the enzyme active site was associated to a peculiarity of the two complexes under investigation, or to a more general behaviour of sulphamate and sulphamide derivatives, a comparative analysis of all hCA II/sulphamate and hCA II/sulphamide structures available in the PDB was undertaken^25^^,^^26^^,^^49–65^^,^^67–71^. Surprisingly, the analysis of all these structures revealed that, independently of the nature of the moiety attached to the ZBG, the distance between the Thr200OG1 atom and the sulphamide nitrogen N2 in hCA II/sulphamide complexes was generally shorter than the corresponding distance between the sulphamate oxygen O3 and the same enzyme atom in hCA II/sulphamate complexes (see Tables 3 and 4). Moreover, in most of the hCA II/sulphamide adducts, such a distance is compatible with the formation of an H-bond, the situation not observed in the case of enzyme/sulphamate complexes.

**Table 3.** Distances between Thr200OG1 atom and the sulphamide N2 atom in hCA II/sulphamide complexes. Only sulphamides of the type R-NH-SO_2_NH_2_ were considered.

Compound	N2-Thr200OG1 distance (Å)	PDB code
	3.2	4MO8
	3.5	2H15
	3.0	3M2X
	3.2	3MNU
	3.0	4MDG
	3.0	4Q78
	3.4	4MDM
	2.9	4MDL
	5.0	4PQ7
	3.7	5FDC
	3.7	5FDI

**Table 4.** Distances between Thr200OG1 atom and the sulphamate O3 atom in hCA II/sulphamate complexes.

Compound	O3-Thr200OG1 distance (Å)	PDB code	Compound	O3-Thr200OG1 distance (Å)	PDB code
	4.7	5O07		3.9	2WD2
	4.1	3IBU		4.0	3IBI
	4.2	3IBN		4.1	3IBL
	4.4	1XQ0		4.8	1XPZ
	4.6	3OIM		3.8	3OKU
	4.3	3T85		4.6	3T82
	5.2	2X7T		5.0	2GD8
	4.4	2X7U		4.8	2X7S
	4.6	4ZWY		4.1	4ZX0
	4.7	3DD8		4.5	1TTM
	4.7	3C7P		4.9	3BET
	4.6	3HKU		4.5	1EOU
	4.1	4ZWI		4.8	4R5B
	4.3	3T84		4.7	3T83
	4.5	2WD3			

**Figure 4.**
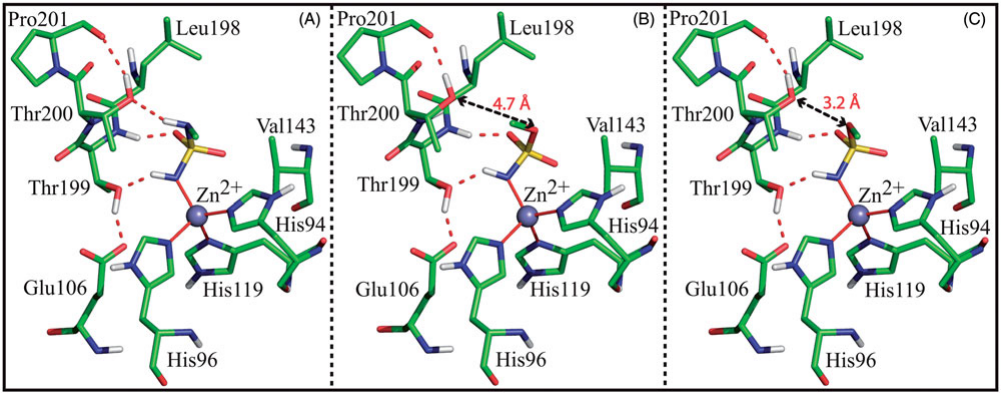
Detail of the active site in the model systems **Complex_N** (A), **Complex_O** (B) and **Complex_NO** (C). In all three cases the ligand, the zinc ion, the three coordinating histidines, Glu106, and enzyme residues giving a major contribution to ligand binding are shown. Only polar hydrogens are shown. Hydrogen bonds are highlighted with red dotted lines, while the distances between O3 and Thr200OG1 are indicated with black arrows.

To understand why the sulphamate oxygen O3 atom was always pushed away from the Thr200OG1 atom with respect to the corresponding atom in sulphamides, binding free energy calculations were carried out. At this aim, the MM/GBSA method, which allows obtaining a per-residue decomposition of the binding free energy, was utilised. To make results independent on the nature of the moiety attached to the ZBG, simplified models of sulphamate/sulphamide derivatives were used. In particular, three model systems, hereafter indicated as **complex_O**, **complex_N** and **complex_NO**, were built. The first two models were obtained starting from the hCA II/**3** and hCA II/**4** crystallographic structures and replacing the 2-methyl-5-nitro-imidazole moiety of the two inhibitors with a methyl group. The third model was obtained by substituting the N2 atom of **complex_N** with an oxygen atom. It is important to highlight that, whereas **complex_O** and **complex_N** represent a simplified version of the hCA II/sulphamate and hCA II/sulphamide crystal structures, **complex_NO** corresponds to a hypothetical hCA II/sulphamate adduct, where the oxygen atom O3 is forced to assume the same position occupied by N2 in hCA II/sulphamide complexes. Before calculations, hydrogen atoms, which were not visible in the crystallographic structures, were added to the models and their positions were energy minimised using the Discover module of InsightII package. It is worth of note that in all the protonated complexes, in agreement with what observed in the neutronic structure of hCA II crystallised at pH 7.5 (PDB code 4Q49)^72^, the hydrogen bound to the Thr200OG1 atom was oriented towards Pro201O atom, in a direction opposite to the position of the ligand (Figure 4). Consequently, the Thr200OG1 atom can act only as a hydrogen bond acceptor when interacting with the ligand. Accordingly, in **complex_N** Thr200OG1 atom establishes a hydrogen bond interaction with the N2 atom of the ligand (Figure 4(A)), which is a hydrogen bond donor. On the contrary, in **complex_O** and **complex_NO**, it cannot form such interaction with O3 atom, since the O3 atom can act only as hydrogen bond acceptor (Figure 4(B,C)).

Table 5 reports results of MM/GBSA calculations, which allowed the identification of all the enzyme residues, beyond the zinc ion, giving a stabilising contribution to the binding of the ligands. Interestingly, in all three model systems four residues, namely Val143, Leu198, Thr199 and Thr200, were identified as major contributors to the binding. Among these, Val143, Leu198, and Thr199 contribute in a similar way in all complexes, whereas Thr200 provides a different contribution to binding free energy in each model, thus confirming the critical role, suggested by crystallographic studies, played by this residue for sulphamate/sulphamide binding. In particular, this residue interacts more favourably with ligand in the case of **complex_N**, showing the lowest value of total binding free energy (Δ*G*_bind_-Thr200 = −3.164 Kcal/mol), whereas it interacts less favourably with ligand in **complex_NO** with a total binding free energy value of −1.290 Kcal/mol. These data can be explained by looking at the individual energy components of Δ*G*_bind_-Thr200 reported in Table 6. Major differences are observed in the contribution of the electrostatic term (Δ*E*_elec_). This term has always a positive value, indicating in all three complexes the presence of unfavourable charge interactions between Thr200 and ligand. Such unfavourable interactions can be mainly ascribed to the repulsion between the partial charges on the backbone nitrogen atom of Thr200 and the N1 atom of the three ligands. Although these atoms are quite far apart in all models (4.5 Å), the energetic calculations probably overestimate their charge repulsion due to the very high negative charge on N1 atom obtained through QM methods (see “Materials and Methods” section). However, since the distance between the backbone nitrogen atom of Thr200 and the N1 atom of the ligand is the same in all three systems, the extent of this repulsive interaction can be considered the same in all of them. Thus, additional contributions have to be considered to explain the observed differences in the electrostatic term. A detailed inspection of the three model systems reveals the presence, in the case of the **complex_NO**, of additional repulsive interactions between the negative partial charges on O3 and Thr200OG1 atoms, which are at a relatively close distance (3.2 Å) (Figure 4(C)), leading to the highest value of Δ*E*_elec_ (2.673 Kcal/mol). In **complex_O**, where the distance between O3 and Thr200OG1 atoms is larger (4.7 Å) (Figure 4(B)), this repulsive electrostatic contribution is significantly reduced ( Δ*E*_elec_ = 1.076 Kcal/mol), thus giving a justification for the preferential binding of sulphamate in this conformation, as observed in crystallographic studies. Finally, in **complex_N**, Δ*E*_elec_ is further reduced (0.526 Kcal/mol) due to the stabilising contribution of the N3-Thr200OG1 hydrogen bond (Figure 4(A)).

**Table 5.** Per-residue binding energy decomposition (given in kcal/mol), calculated by the MM/GBSA method for **complex_N**, **complex_NO** and **complex_O**. Only residues contributing more than −1.0 kcal/mol to the binding are reported.

	Δ*G*_bind_-Val143	Δ*G*_bind_-Leu198	Δ*G*_bind_-Thr199	Δ*G*_bind_-Thr200
**complex_N**	−1.224	−5.536	−1.409	−3.164
**complex_NO**	−1.177	−5.467	−1.604	−1.290
**complex_O**	−1.625	−5.209	−1.764	−2.007

**Table 6.** Individual energy components (kcal/mol) of Δ*G*_bind_-Thr200 calculated by the MM/GBSA method for **complex_N**, **complex_NO** and **complex_O**.

	Δ*E*_vdW_^[a]^-Thr200	Δ*E*_elec_^[b]^-Thr200	Δ*G*_GB_^[c]^-Thr200	Δ*G*_sur_^[d]^-Thr200
**complex_N**	−1.430	0.526	−1.249	−1.011
**complex_NO**	−1.362	2.673	−1.716	−0.885
**complex_O**	−1.002	1.076	−1.221	−0.860

[a] van der Waals contribution.

[b] Electrostatic contribution.

[c] Generalised-Born solvation contribution.

[d] Non-polar solvation contribution.

In conclusion, energetic calculations showed that in the crystallographic structures of hCA II/sulphamate adducts the O3 sulphamate oxygen atom prefers to be placed in a position more distant from the Thr200OG1 atom with respect to the corresponding N2 atom in hCA II/sulphamide complexes, in order to reduce unfavourable electrostatic interactions.

## Conclusions

Sulphamates and sulphamides derivatives have been largely investigated as CAIs^1^^,^^14^^,^^15^ by means of different experimental techniques. However, the structural determinants responsible for their different binding mode to the enzyme active site were not clearly defined so far. In this paper, we report a combined crystallographic and theoretical study on these compounds, demonstrating that electrostatic interactions with residues within the enzyme active site play a key role in determining the binding conformation of these molecules. Due to these interactions, molecules that differ only for one atom, as in the case of compounds **3** and **4**, can assume a completely different orientation within the CA active site. A similar situation was observed also in the case of topiramate **1** and its sulphamide analogue **5** (see Figure 1). Indeed, also in this case, a single atom substitution creates differences in the arrangement of the organic scaffold with the CA II active site, and consequently in *K_I_* values against the enzyme^69^. These findings open new important perspectives in the field of CAI drug design. Indeed, as mentioned in the ‘Introduction’ section, in the past sulphamide derivatives were considered not particularly suitable for obtaining potent CAIs, mainly due to lower acidity of the sulphamide group with respect to sulphamate one and to the lower tendency to form the anionic form required for CA inhibition^22^. The study here reported demonstrates that other factors can play a key role in determining the affinity of sulphamide/sulphamate derivatives for the CA active site and that, as observed for compounds **3** and **4**, these factors can also lead to a higher affinity of sulphamide derivatives with respect to the corresponding sulphamates for CAs.
